# A general form of capillary rise equation in micro-grooves

**DOI:** 10.1038/s41598-020-76682-2

**Published:** 2020-11-12

**Authors:** Gholamreza Bamorovat Abadi, Majid Bahrami

**Affiliations:** Laboratory for Alternative Energy Conversion (LAEC), School of Mechatronic Systems Engineering, Simon Fraser University, Burnaby, BC V3T 0A3 Canada

**Keywords:** Energy science and technology, Engineering, Mathematics and computing, Physics

## Abstract

Micro-grooves are a crucial feature in many applications, such as microelectro-mechanical systems, drug delivery, heat pipes, sorption systems, and microfluidic devices. Micro-grooves utilize capillary action to deliver a liquid, with no need for an extra pumping device, which makes them unique and desirable for numerous systems. Although the capillary action is well studied, all the available equations for the capillary rise are case-specific and depend on the geometry of the groove, surface properties, and the transport liquid. In this study, a unified non-dimensional model for capillary rise is proposed that can accurately predict the capillary rise for any given groove geometry and condition and only depends on two parameters: contact angle and characteristic length scale, defined as the ratio of the liquid–vapor to the solid–liquid interface. The proposed model is compared against data from the literature and can capture the experimental results with less than 10% relative difference. The effect of the grooves’ height, width, and contact angle is investigated and reported. This study can be used for a unified approach in designing heat pipes, capillary-assisted evaporators for sorption systems, drug delivery micro-fluidic devices, etc.

## Introduction

The self-driving flow of a liquid in a capillary micro-groove is an important feat of engineering with a wide range of applications, from space applications due to microgravity to power electronics and heat pipes, to sorption technology and capillary-assisted evaporators.

One main application of capillary action in micro-grooves is in heat pipes. These two-phase heat transfer devices are a crucial part of any modern power electronic device. In fact, the capillary rise is one of the important factors in designing heat pipes. Hopkins et al.^1^ experimented with flat miniature heat pipes to determine the maximum heat flow rate and heat flux for different operating temperatures. They concluded that heat pipes with deep and narrow capillary grooves produce the best results while most of their data showed that the heat flux bottleneck of the evaporator was the capillarity limitation. Ma and Peterson^2^ performed a series of experiments to determine the heat transport in triangular grooves, similar to those used in heat pipes, purely based on the capillary rise limit. They reported the maximum capillary heat transport capacity and pure capillary limit of methanol flowing in triangular grooves. Another example of capillary limit in heat pipes is^3^. They concluded that a heat pipe’s performance is greatly dominated by the capillary limit. They established a mathematical model of the capillary limit for a micro heat pipe with trapezium-grooved substrate.

Vapor chambers or flat heat pipes are of particular interest and are used extensively in electronic devices due to their reliability, simplicity, passive operational mode, and effective heat transport capacity^4^. They remove the need for active liquid-cooling while providing a high-performance heat removal capability. Weibel and Garimella^4^ note that the high performance of heat pipes and vapor chambers depends on the capillary pressure generated by the wick material so that it can overcome the viscous and inertial pressure drops along the vapor and liquid flow paths.

A general understanding of flow in open micro-grooves and its limitation is available in the literature for different geometries. Zhang et al.^5^ explored the mechanism of open channel capillary flow experimentally and numerically, with applications such as the refueling stations of the International Space Station (ISS). They calculated the critical flow rate and the height of fluid level using the Newton method. Similarly, Haake et al.^6^ investigated the liquid flow through open capillary grooves experimentally and numerically. They also concluded that there exists a capillary flow limit. Other notable works on capillary-driven flow in open grooves can be found in^7–13^. More recently, attention has been given to surface properties and its effect on capillary rise. Kim et al.^14^ experimented on hydrophilic surfaces to observe the capillary rise dynamics within channels and concluded that the capillary rise is initially governed by the bulk rise.

In sorption cooling and heat pump technology, the main obstacle preventing commercialization is size and weight. Capillary-assisted low-pressure evaporators (CALPEs) are used in closed-cycle sorption systems, including heat pumps, heat transformers, desalination, and thermal energy storage systems^15^. A CALPE eliminates the need for a circulating pump in the low-pressure evaporator, taking advantage of the capillary phenomena. After experimenting with a series of enhanced heat transfer tubes featuring circumferential rectangular micro-grooves, liquid height due to capillarity, evaporation pressure, and the degree of superheat were deemed the most important factors in heat transfer performance^15,16^.

From this introduction, two points are concluded: (1) the capillary action in micro-grooves have a wide range of application. In most of these applications, the capillary rise or the ability of the groove or a wicking material to transport the liquid is the design bottleneck; and (2) all the experimental, analytical, or numerical studies dealing with this topic are case-specific and can be applied to only a specific geometry or transport liquid and cannot be generalized. The objective of this paper is to provide an analytical solution to the capillary rise in micro-grooves by using a fundamental approach and to propose a unified equation. There is a desire to develop an analytical model that can predict the capillary rise in any groove with a given cross-section, since it removes extra calculation steps and unifies the capillary equation regardless of the cross-sectional area. It is understandable that there is a need for exploring the fundamentals of capillarity, capillary rise equations, and the important parameters affecting it to reach a unified approach. In this study, the capillary rise in micro-grooves with selected cross-sections (rectangular, cylindrical, curved, trapezoidal, triangular, and hyperellipse) is studied analytically, the relative importance of different parameters (contact angle, groove width, depth, etc.) is investigated, and then a novel, general, and non-dimensional equation is proposed.

## The capillary rise equation for various cross-sections

Here, we start with a rectangular cross-section groove, list the assumptions, provide the governing equations, and make a conclusion on the capillary rise equation. Similar steps can be taken for other cross-sections. The final results for all the studied cross-sections are summarised in Table 1 and the detailed step-by-step procedure is given in Appendix I. The assumptions are as follows:

- The open grooves’ width is small enough for the capillary action to occur, but is not too small (length scale > 10 nm), therefore nanoscale effects are negligible^17^
- Partial capillarity is not studied, and it is assumed that the full area of the grooves is filled with a liquid (e.g. water),
- The micro-groove is placed vertically (or with a slanted angle) so that the bottom end always touches a big fluid reservoir,
- The physical properties of all materials are constant,
- The vapor–liquid interface is homogenous, and
- Heat transfer is negligible since the capillary action is a fast, almost instantaneous process.

**Table 1:** Capillary rise equation for various cross-sections.

Capillary rise equation	Groove cross-section	Non-dimensional equation	References
$h_{rec} = \frac{{\sigma \left[ {\left( {2D + W} \right)cos\theta - W} \right]}}{\rho gDW Sin\alpha }$ ${\text{P}}_{{\text{w}}} = 2{\text{D}} + {\text{W}}$ ${\text{A}}_{{\text{c}}} = {\text{D}} \times {\text{W}}$		$h_{rec}^{*} = cos\theta - \frac{W}{{P_{w} }}$	^21^
$h_{{cyl}} = \frac{{\sigma \left[ {\left( {\pi R + 2d} \right)cos\theta - 2R} \right]}}{{\rho g\left[ {2dR + \pi R^{2} /2} \right]Sin\alpha }}$ ${\text{P}}_{{\text{w}}} = \pi R + 2d$ ${\text{A}}_{{\text{c}}} = 2dR + \pi R^{2} /2$		$h_{cyl}^{*} = cos\theta - \frac{W}{{P_{w} }}$	^23^
$h_{{tri}} = \frac{{\sigma \left[ {2cos\theta \sqrt {D^{2} + \frac{{W^{2} }}{4}} - W} \right]}}{{\rho gWD/2~Sin\alpha }}$ ${\text{P}}_{{\text{w}}} = 2\sqrt {D^{2} + \frac{{W^{2} }}{4}}$ ${\text{A}}_{{\text{c}}} = W \times D/2$		$h_{tri}^{*} = cos\theta - \frac{W}{{P_{w} }}$	^24^
$h_{{curv}} = \frac{{\sigma \left[ {\left( {\pi R + 2d + l} \right)cos\theta - 2R - l} \right]}}{{\rho g\left[ {2dR + dl + Rl + \pi R^{2} /2} \right]Sin\alpha }}$ ${\text{P}}_{{\text{w}}} = \pi R + 2d + l$ ${\text{A}}_{{\text{c}}} = 2dR + l\left( {d + R} \right) + \pi R^{2} /2$		$h_{curv}^{*} = cos\theta - \frac{W}{{P_{w} }}$	^23^
$h_{{trap}} = \frac{{\sigma \left[ {\left( {l + 2\sqrt {D^{2} + \frac{{\left( {W - l} \right)^{2} }}{4}} } \right)cos\theta - W} \right]}}{{\rho g\left( {l + W} \right)D/2~Sin\alpha }}$ ${\text{P}}_{{\text{w}}} = l + 2\sqrt {D^{2} + \frac{{\left( {W - l} \right)^{2} }}{4}}$ ${\text{A}}_{{\text{c}}} = \left( {l + W} \right) \times D/2$		$h_{trap}^{*} = cos\theta - \frac{W}{{P_{w} }}$	–
$h_{{ellip}} = \frac{{\sigma \left[ {\left( {2bE\left( {\sqrt {1 - \varepsilon ^{2} } } \right)} \right)cos\theta - 2b\varepsilon } \right]}}{{\rho g\left[ {\pi \varepsilon b^{2} /2} \right]Sin\alpha }}$ ${\text{P}}_{{\text{w}}} = 2bE(\sqrt {1 - \varepsilon^{2} } )$ ${\text{A}}_{{\text{c}}} = \pi \varepsilon b^{2} /2$		$h_{ellip}^{*} = cos\theta - \frac{W}{{P_{w} }}$	^25^

**Figure 1:**
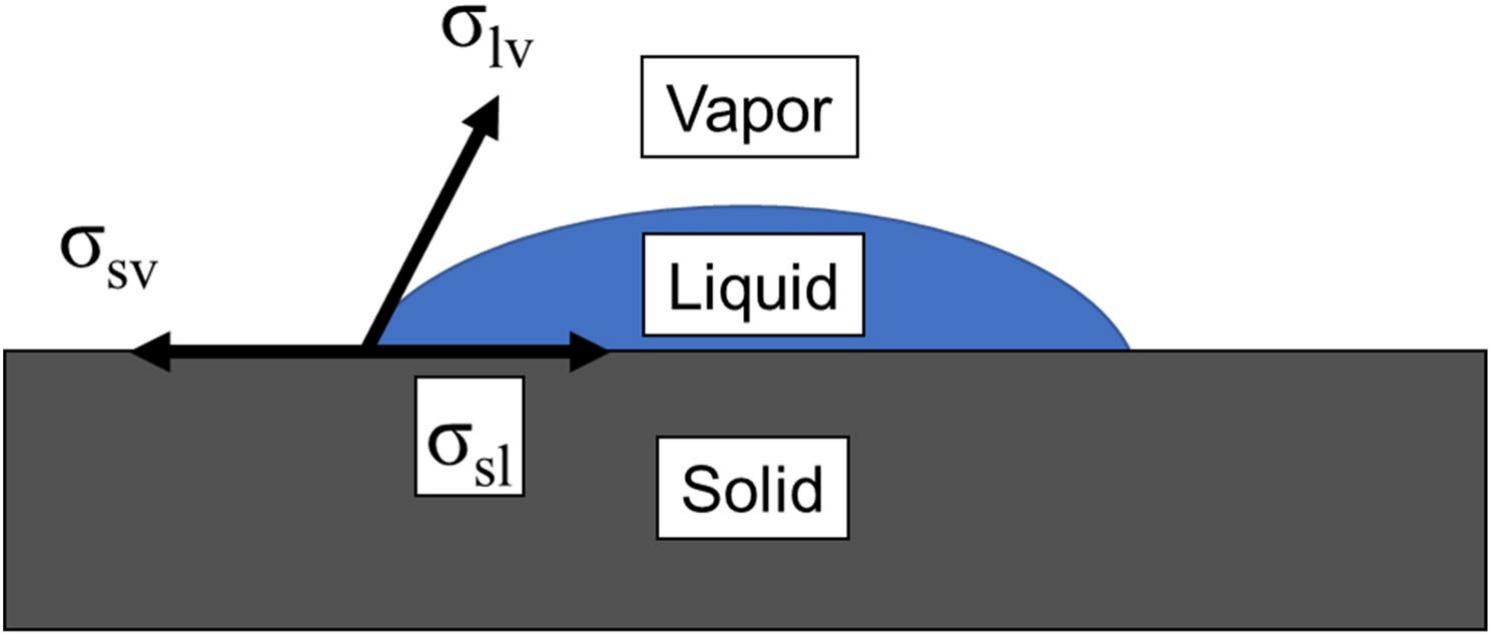
A schematic of a drop of a liquid on a flat surface showing three forces of surface tension leading to Young’s equation under static equilibrium.

**Figure 2:**
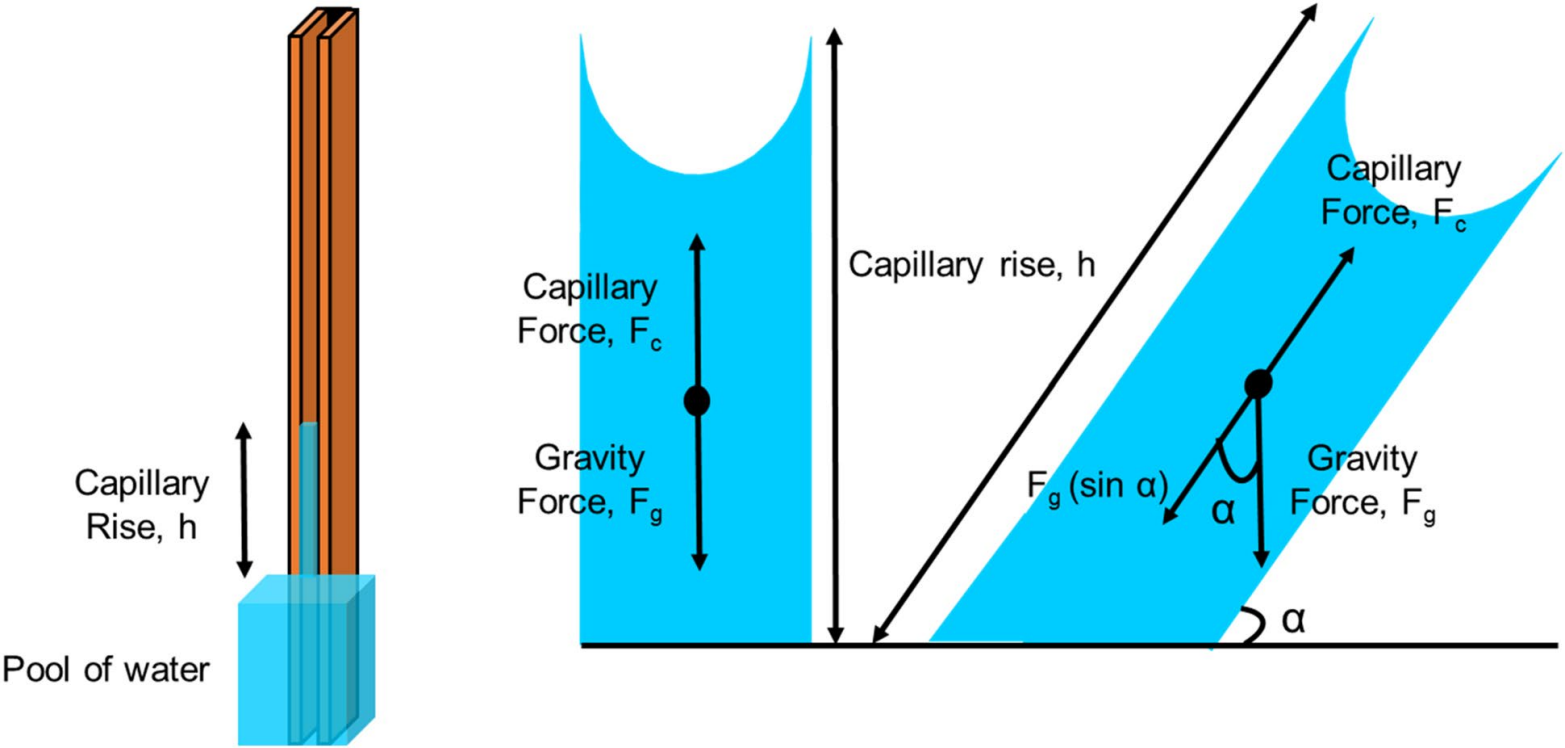
Top and front view of a rectangular micro-groove.

**Figure 3:**
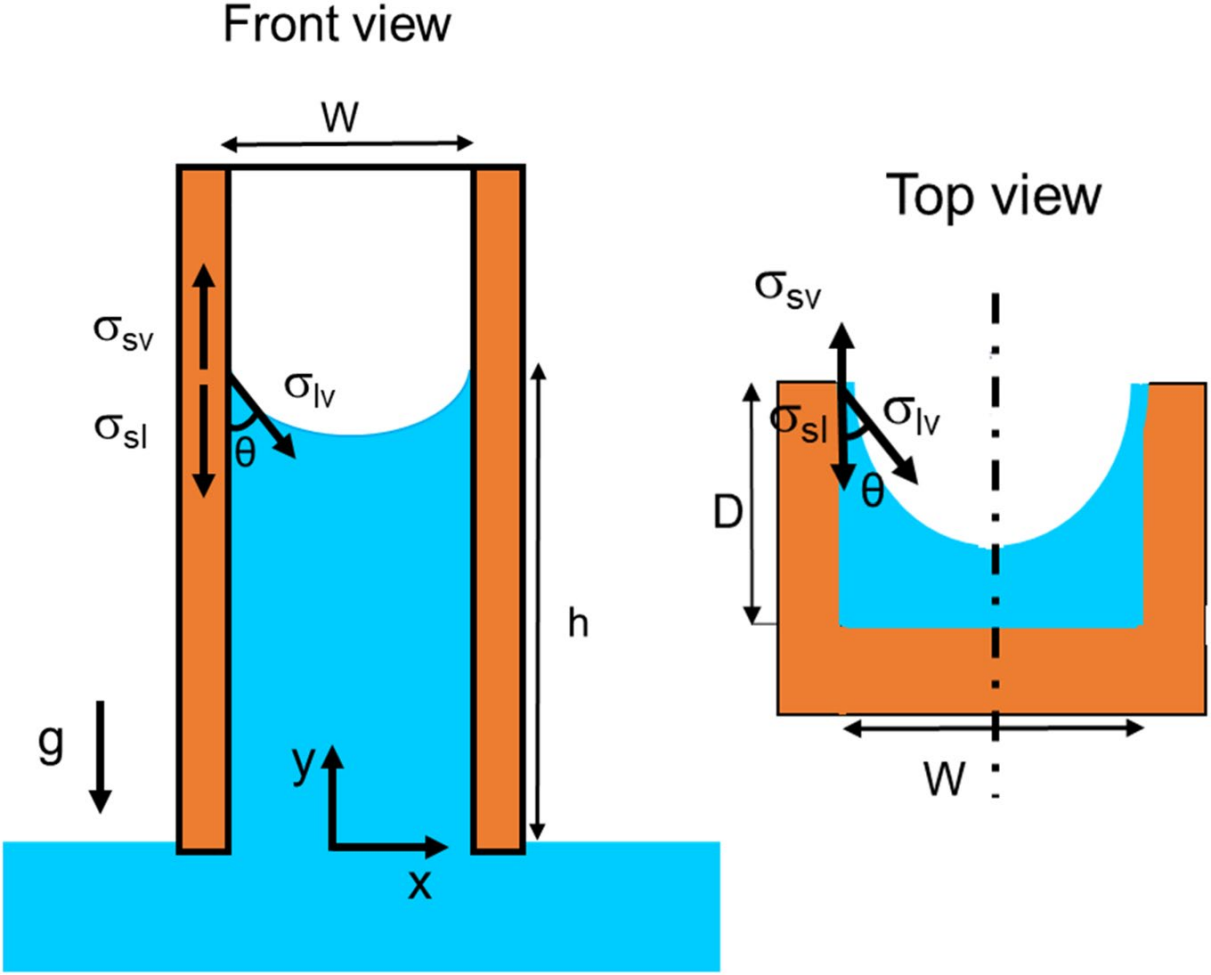
Top and front view of a rectangular micro-groove.

Considering these assumptions, and looking at Fig. 1, the Young’s equation can be written as follows^18–20^:

1 $$\upsigma _{{{\text{sv}}}} =\upsigma _{{{\text{sl}}}} +\upsigma _{{{\text{lv}}}} \cos \,\uptheta$$

here, *σ* and *θ* are surface tension and contact angle, respectively. When a micro-groove with a rectangular cross-section is placed in a liquid as shown in Fig. 2, the change in interface area of the liquid–vapor and solid–liquid are respectively:

2 $${\text{dA}}_{{{\text{lv}}}} = {\text{Wdy}}$$

3 $${\text{dA}}_{{{\text{sl}}}} = \left( {2{\text{D}} + {\text{W}}} \right){\text{dy}}$$

where, *D* is the groove depth and *W* is the grove width. Here, to further generalize the equations, a slanted column is considered that is tilted with the angle *α*. Therefore, in general form, the micro-groove is not vertical unless *α* = 90, as seen in Fig. 2. Figure 3 shows more details of front and top view of the micro-groove, the surface tension vectors, and the direction of gravity.

Looking at Fig. 3, the Helmholtz free energy between three interfaces can be written as^21,22^:

4 $${\text{dE}} =\upsigma _{{{\text{sv}}}} {\text{dA}}_{{{\text{sv}}}} +\upsigma _{{{\text{sl}}}} {\text{dA}}_{{{\text{sl}}}} +\upsigma _{{{\text{lv}}}} {\text{dA}}_{{{\text{lv}}}}$$

The capillary force is given as “-dE/dy”. It follows that:

5 $${\text{F}}_{{\text{c}}} = - {\text{dE}}/{\text{dy}} =\upsigma _{{{\text{lv}}}} [\left( {2{\text{D}} + {\text{W}}} \right)\cos \,\uptheta - {\text{W}}]$$

The capillary force balances the gravity force that is exerted on the weight of the water column, resulting in the following capillary rise equation for a rectangular cross-section groove^21^:

6 $$h_{rec} = \frac{{\sigma \left[ {\left( {2D + W} \right)cos\theta - W} \right]}}{\rho gDW Sin\alpha }$$

Appendix I provides a detailed and step-by-step approach of how Eq. 6 is concluded. It also explores other cross-sectional areas and how the capillary rise equation resulted for them.

Following the same approach, the capillary rise equation can be derived for any cross-section. Table 1 lists the capillary rise equation for six cross-sections of rectangular^21^, cylindrical^23^, curved (modified form of^23^), trapezoidal, triangular, and general form of hyperellipse. The effect of groove’s width, height, wetting perimeter (defined later), contact angle, and surface tension can be studied using Eq. (6).

## The unified non-dimensional capillary rise equation

An analytical model that can predict the capillary rise in any cross-section groove is highly desired since it removes extra steps and unifies all the equations in Appendix I. Equation 6 can be non-dimensionalized by rearranging and introducing two parameters: *P*_*w*_ is the wetting perimeter and denotes the wetted length of the cross-section (excluding the liquid–vapor interface; in other words, the wetting perimeter is the solid–liquid interface length), and *A*_*c*_ is the cross-sectional area. For a rectangular cross-section, *P*_*w*_ and *A*_*c*_ are given as:

7 $${\text{P}}_{{\text{w}}} = 2{\text{D}} + {\text{W}}$$

8 $${\text{A}}_{{\text{c}}} = {\text{DW}}$$

For a rectangular cross-section, the non-dimensional capillary rise is:

9 $$h^{*} = \frac{\rho gh Sin\alpha }{\sigma }*\frac{{A_{c} }}{{P_{w} }} = cos\theta - \frac{W}{{P_{w} }}$$

where *L* $= \frac{W}{{P_{w} }}$ is a characteristic length scale and is the ratio of the liquid–vapor interface to the wetting perimeter. Appendix II demonstrates the step-by-step procedure to non-dimensionalize Eq. 6 and conclude Eq. (9). Looking at the capillary rise equations in the Appendix I and using *P*_*w*_, it is concluded that in fact all the Eqs. A.9, 18, 27, 36, 45, and 54 can be written similarly to Eq. (9). This fact is given in Table 1 where all the non-dimensional capillary rise equations are listed along with the cross-sectional area and wetting perimeter for each cross-section.

## Results and discussion

**Figure 4:**
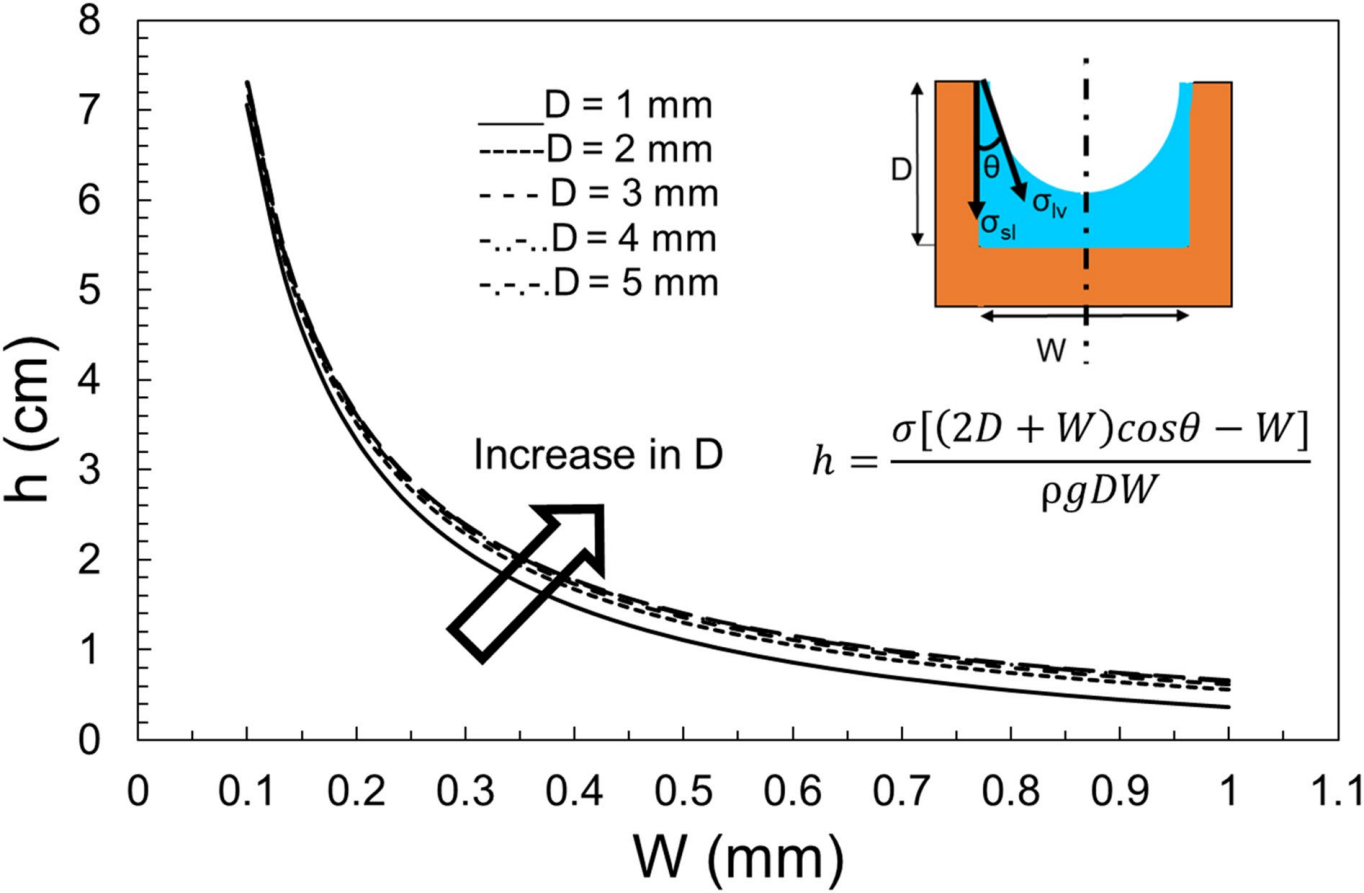
The effect of groove width and height on the capillary rise in a rectangular cross-section for α = 90°.

**Figure 5:**
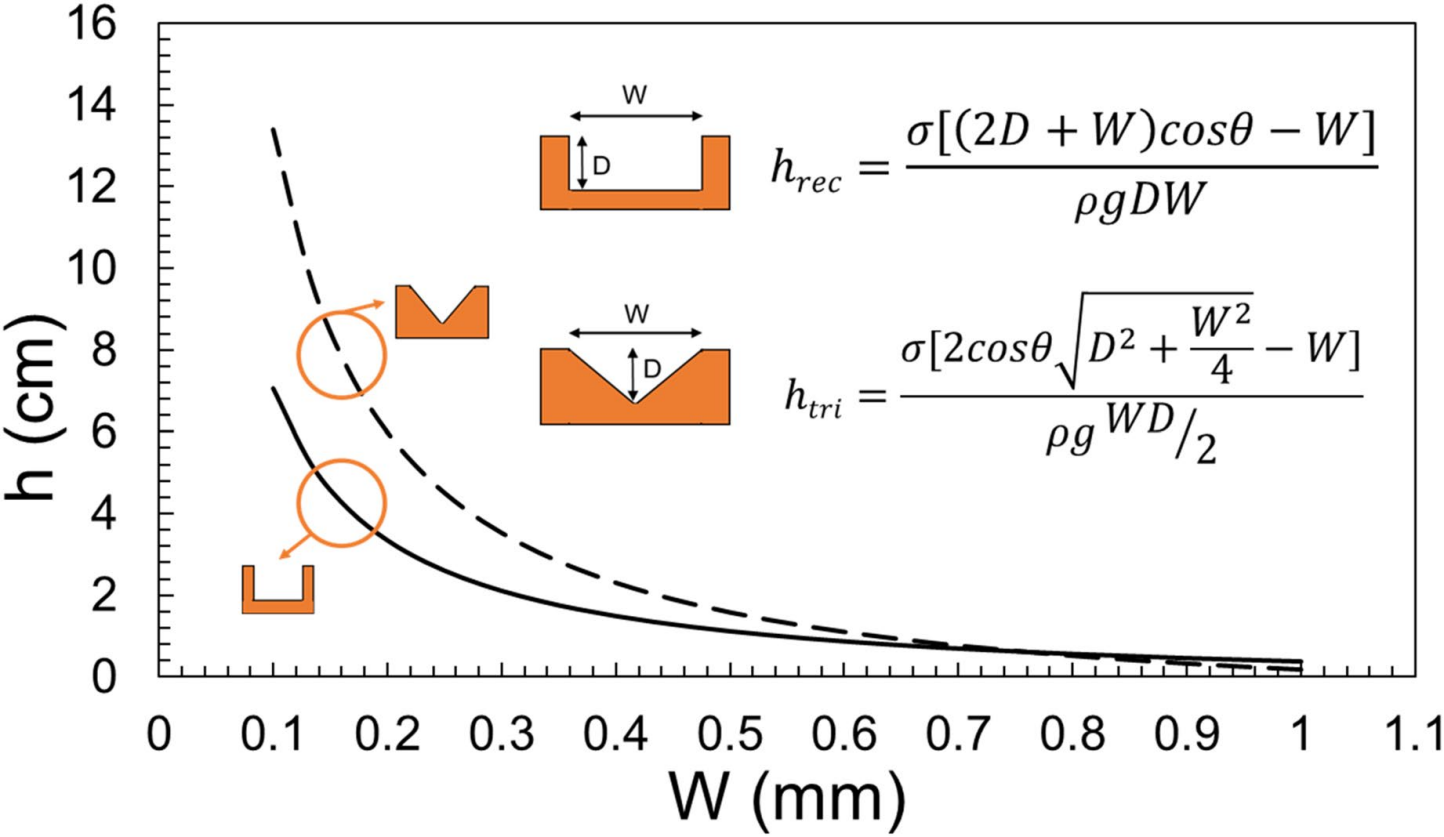
A comparison between the capillary rise in a rectangular and triangular cross-section groove, D = 1 mm, and α = 90°.

The effect of the groove’s width, height, wetting perimeter, contact angle, and surface tension can be all studied using Eq. (6). Although, most of the upcoming plots are for a rectangular groove, a similar approach and conclusions can be made for various cross-sections. Before plotting the non-dimensional parameters, the dimensional capillary rise is plotted for different cross-sections. Figure 4 shows the effect of depth and width on capillary height of a rectangular groove. It is concluded that the change in width of the groove has a greater effect on capillary height than the change in depth. Figure 5 presents a comparison between the capillary rise in a rectangular and triangular groove. It is seen that the capillary rise in a triangular groove is considerably higher than that in a rectangular cross-section. This fact is explained by the fact that a triangular groove contains half of the liquid as compared to a rectangular one, therefore, the capillary force is able to take the body of water higher.

**Figure 6:**
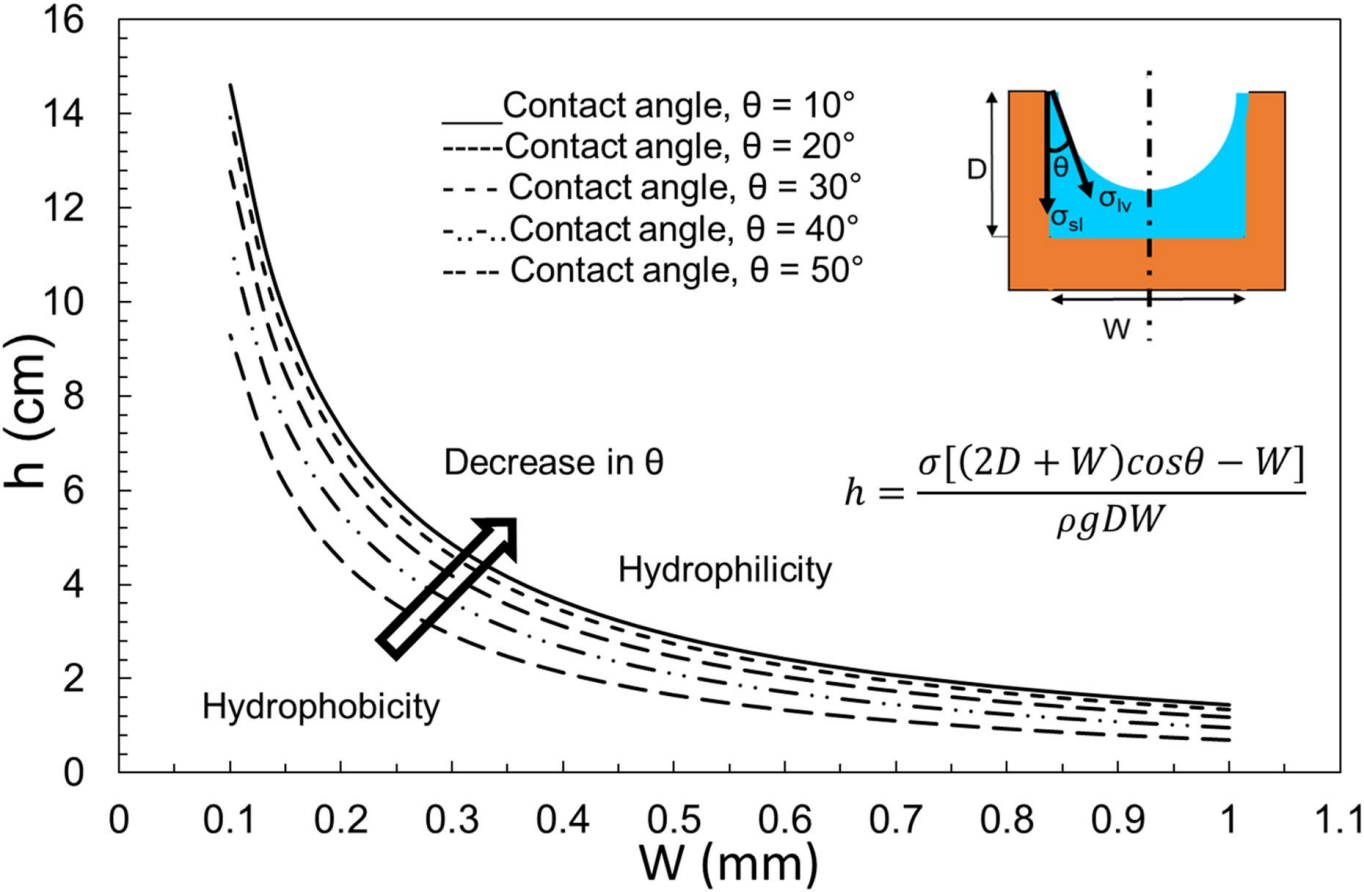
The effect of contact angle on the capillary rise in a rectangular cross-section with D = 1 mm and α = 90°.

**Figure 7:**
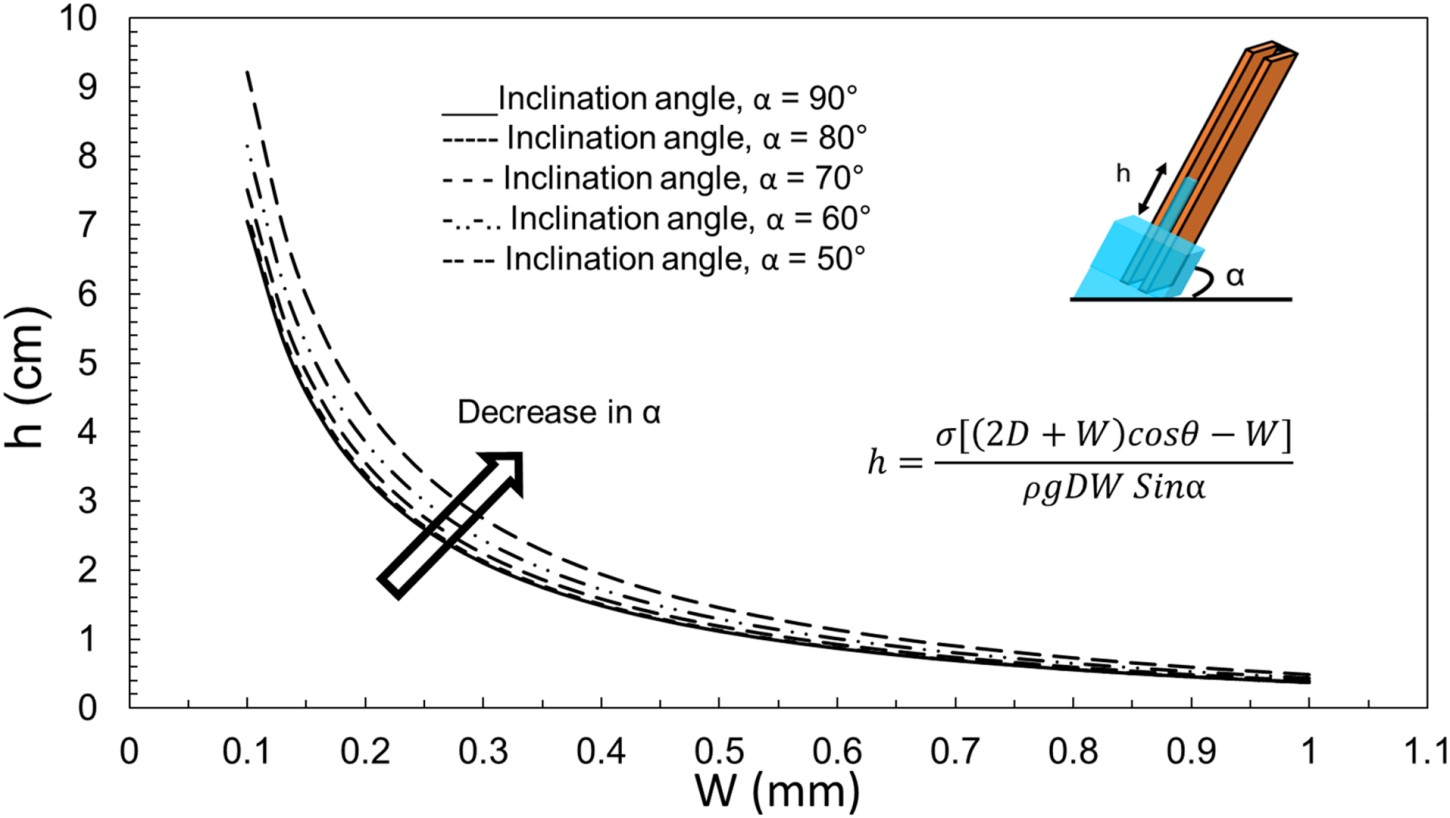
The effect of inclination angle on the capillary rise in a rectangular cross-section with D = 1 mm.

The contact angle has a great effect on the overall capillarity in micro-grooves. Figure 6 plots the effect of contact angle on capillary height in a rectangular groove. It is concluded that the more hydrophilic the surface, the higher the capillary rise would be. Although, Fig. 4 was plotted for an inclination angle of 90°, it is possible to investigate the effect of α. Figure 7 plots the capillary height as the inclination angle drops from 90 to 40 degrees. It is worth mentioning that for α smaller than 90°, *h* is the capillary length, the length of rise of the liquid along the groove, rather than the overall height of the liquid column (refer to Fig. 3 for clarification). It is concluded that for smaller inclination angles, the liquid travels further to reach the same water height level as a vertical groove (since the pressure of the pool of water and that of the top of the capillary height should equalize).

**Figure 8:**
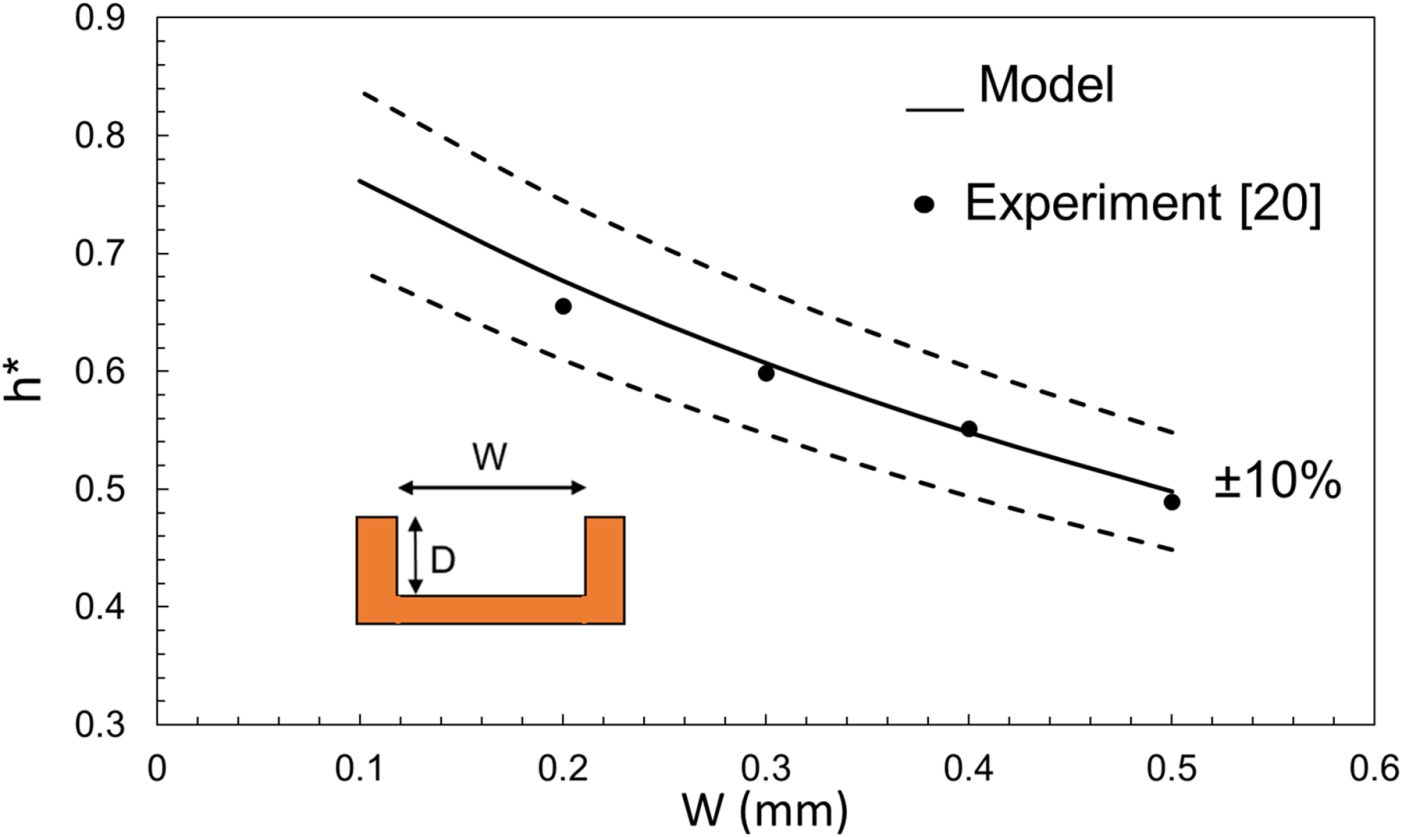
A comparison of capillary rise (Eq. 9) with data extracted from^21^.

**Figure 9:**
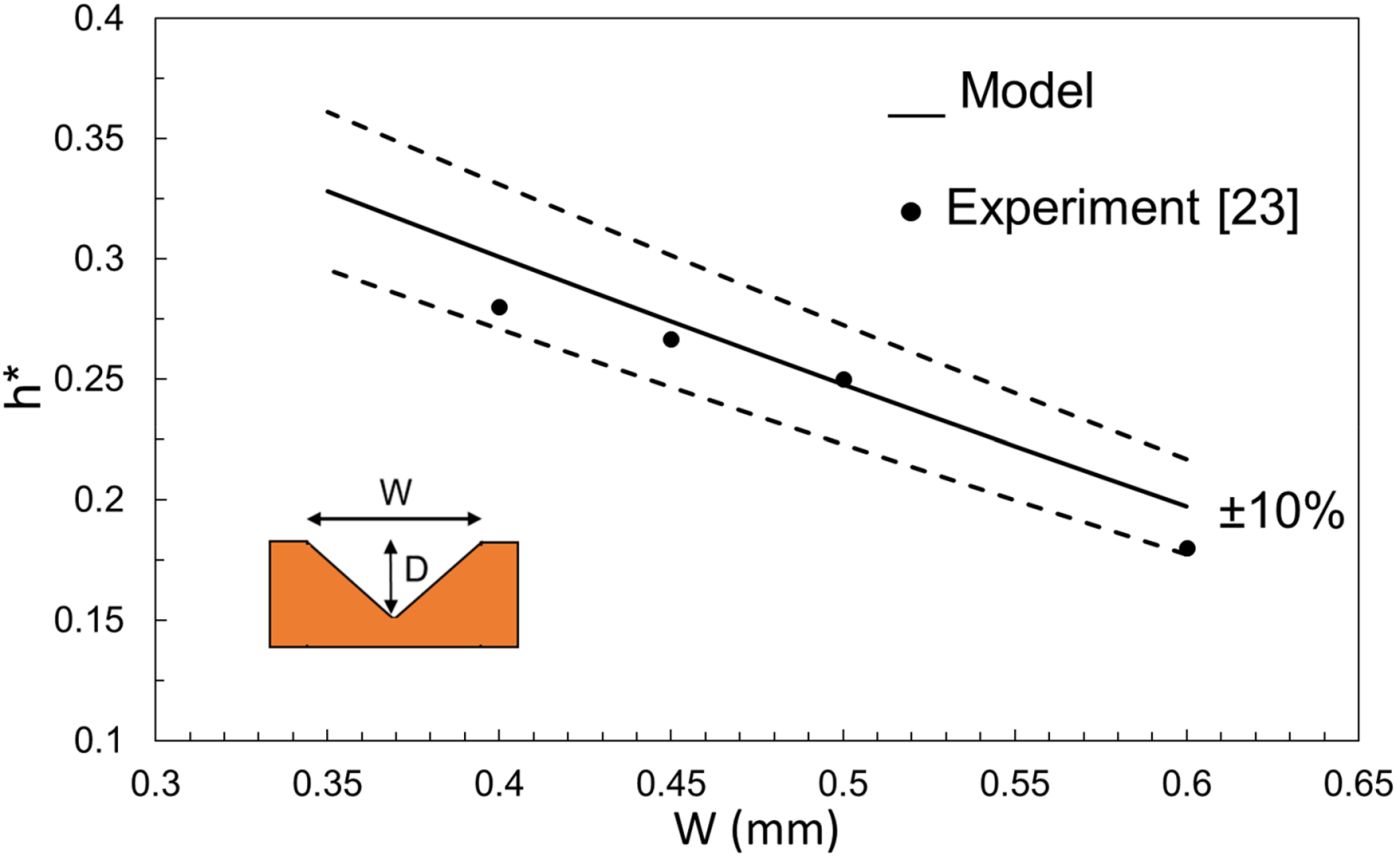
A comparison of capillary rise (Eq. 9) with data extracted from^24^.

The unified non-dimensional capillary rise is given as Eq. (9). Before plotting a general form of this equation, it is interesting to compare the proposed model with experimental data from the literature. Here, two sets of data from^21^ and^24^ are chosen to see how Eq. 9 can capture the experimental capillary rise in a non-dimensional form. Figures 8 and 9 compare the predicted h^*^ for a rectangular and triangular cross-section groove, respectively, with those from the literature. It is seen that the non-dimensional form of *h* falls within 10% of the experimental data, therefore, it can be used as a general and unified equation. The reported deviation of experimental data from the model’s values is typical and expected, given many factors that affect a capillary rise experiment. Possible sources of this deviation are surface roughness, contact angle variation, and geometry inconsistency^21^.

**Figure 10:**
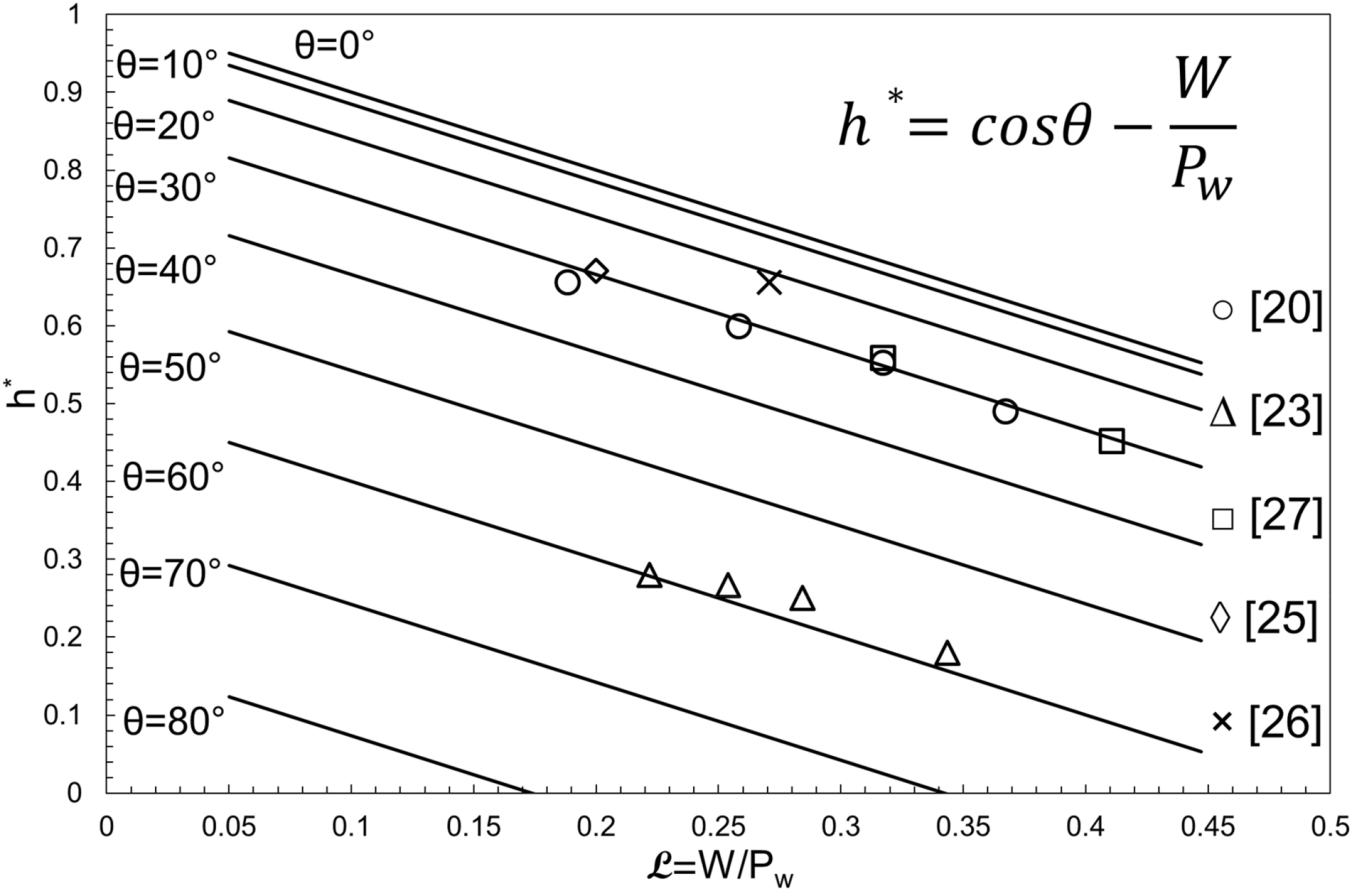
The unified non-dimensional form of capillary rise as a function of characteristic length scale and contact angle. Data from (○^21^, ∆^24^, □^28^, ◊^26^, × ^27^).

Figure 10 plots the final unified non-dimensional form of capillary rise as a function of *L*, characteristic length scale. It is seen that for each value of the contact angle, h^*^ is a line that intercepts the x-axis at some point. Returning to the first assumption in “The capillary rise equation for various cross-sections” section, the maximum allowable characteristic length scale depends on the contact angle value; i.e. there exists a maximum *L,* for each contact angle value, above which the capillary action would not happen for that contact angle. In other words, for capillary to happen (h^*^ > 0), the characteristic length scale should be smaller than a certain value. This value depends on the contact angle; i.e. with a given contact angle, there exists a maximum characteristic length scale (hence, a maximum width), above which h^*^ would be zero. As an example, if contact angle is 70°, the maximum possible characteristic length scale (W/P_w_) is ~ 0.34 (Fig. 10). Therefore, by having the P_w_ of the channel, we can find a maximum width for capillary to happen.

It is also seen that surfaces with a smaller contact angle (more hydrophilic) would have a higher h^*^. Finally, it is concluded that the difference between two sets of h^*^ lines increases significantly as the contact angle increases; i.e. The h^*^ lines for 0° and 10° contact angles are a lot closer to each other than the lines for 50° and 60°, even though they both have a 10° difference in contact angle. Figure 10 also includes experimental data from Refs.^21,24,26,27^ and numerical data from^28^ for reference.

## Conclusions

In this study, a unified non-dimensional closed-form analytical solution was proposed that can accurately predict the capillary rise for any given geometry, and only depends on two parameters: contact angle and a characteristic length scale, defined as the ratio of the liquid–vapor to solid–liquid interface. The effect of a groove’s width, height, wetting perimeter, contact angle, and surface tension were studied using Eq. (6). It was observed that:

- The change in width of the groove had a greater effect on capillary height than the change in depth,
- The capillary rise in a triangular groove was considerably higher than for other cross-sections,
- The contact angle had a great effect on the overall capillarity in micro-grooves. The more hydrophilic the surface, the higher the capillary rise, and
- Smaller inclination angles led to a longer liquid travel path.

It was seen that the unified non-dimensional form of *h* falls within 10% of the experimental data, therefore, it can be used as a general equation. It was also observed that:

- Using the proposed model, Eq. (9), a maximum characteristic length scale, *L*, for applicability of capillary action can be found for a given contact angle,
- Surfaces with smaller contact angle (more hydrophilic) would have a higher h^*^, and
- The difference between two sets of h^*^ lines increases significantly as the contact angle increases.

This general approach can be used as a unifying tool for designing various engineering solutions that involves any micro-groove.

## Supplementary information

Supplementary Information.

## Data Availability

All data generated or analysed during this study are included in this published article and the supplementary materials.

## List of symbols

*A*: Area (m^2^)
*D*: Depth (m)
*F*: Force (N)
*g*: Gravitational acceleration (m/s^2^)
*h*: Capillary rise (m)
*L*: Characteristic length scale (m)
*P*_*w*_: Wetting perimeter (m)
*R*: Radius (m)
*W*: Width (m)
*α*: Inclination angle (°)
*θ*: Contact angle (°)
*ρ*: Density (kg/m^3^)
*σ*: Surface tension (N/m)

## Subscripts

*sv*: Solid–vapor
*sl*: Solid–liquid
*lv*: Liquid–vapor
*c*: Capillary
