# Supplementary material for: A general form of capillary rise equation in micro-grooves
Source: Sci Rep. 2020 Nov 12;10:19709. doi: 10.1038/s41598-020-76682-2 (PMC7665055; doi:10.1038/s41598-020-76682-2)
Supplement: Supplementary file 1 — Supplementary Information. [file 41598_2020_76682_MOESM1_ESM.docx]

A general form of capillary rise equation in micro-grooves

Gholamreza Bamorovat Abadi^1*^ and Majid Bahrami^1^

*^1^Laboratory for Alternative Energy Conversion (LAEC), School of Mechatronic Systems Engineering,* Simon Fraser University, BC, Canada V3T 0A3, [gbamorov@sfu.ca](mailto:gbamorov@sfu.ca), mbahrami@sfu.ca

# Appendix I

In this Appendix, a step-by-step approach is given to attain the final capillary rise equation for different cross sections.

*Rectangular cross-section:*
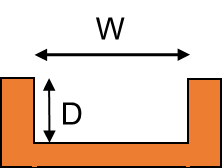


σ_sv_ = σ_sl_ + σ_lv_ cos θ (A.1)

F_g_= mg=ρg(DWh) (A.8)

F_c_= -dE/dy=σ_lv_[(2D+W)cosθ-W] (A.7)

dA_sl_=(2D+W)dy (A.6)

dA_lv_=Wdy (A.5)

E=σ_lv_(A_lv_-A_sl_cosθ) (A.4)

F_c_= -dE/dy (A.2)

dE=σ_sv_dA_sv_ + σ_sl_dA_sl_+ σ_lv_dA_lv_ (A.3)

Since F_c_=F_g_ $Sin\alpha$, it results:

| $h_{rec}=\frac{\sigma[\left( 2D+W \right)cos\theta-W]}{\rho gDW Sin\alpha}$ | (A.9) |
| --- | --- |

*Cylindrical cross-section:*
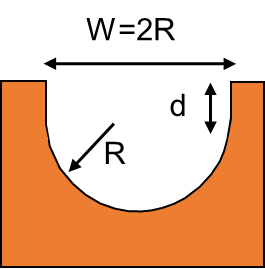


σ_sv_ = σ_sl_ + σ_lv_ cos θ (A.10)

F_g_= mg =ρgh[2dR+0.5πR^2^] (A.17)

F_c_= -dE/dy = σ_lv_[-2R+(2d+πR)cosθ] (A.16)

dA_sl_ =(2d+πR)dy (A.15)

dA_lv_= 2Rdy (A.14)

E=σ_lv_(A_lv_-A_sl_cosθ) (A.13)

F_c_= -dE/dy (A.11)

dE=σ_sv_dA_sv_ + σ_sl_dA_sl_+ σ_lv_dA_lv_ (A.12)

Since F_c_=F_g_ $Sin\alpha$, it results:

| $h_{cyl}=\frac{\sigma[(\pi R+2d)cos\theta-2R]}{\rho g\left[ 2dR+\frac{\pi R^{2}}{2} \right]Sin\alpha}$ (A.18) |
| --- |

*Triangular cross-section:
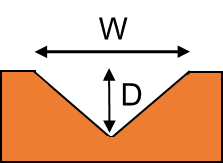
*

σ_sv_ = σ_sl_ + σ_lv_ cos θ (A.19)

F_g_= mg= ρg(DWh/2) (A.26)

F_c_= -dE/dy =σ_lv_[(2 cosθ √(𝐷^2+𝑊^2/4) -W] (A.25)

dA_sl_ =2√(𝐷^2+𝑊^2/4)dy (A.24)

dA_lv_= Wdy (A.23)

E=σ_lv_(A_lv_-A_sl_cosθ) (A.22)

F_c_= -dE/dy (A.20)

dE=σ_sv_dA_sv_ + σ_sl_dA_sl_+ σ_lv_dA_lv_ (A.21)

Since F_c_=F_g_ $Sin\alpha$, it results:

| $h_{tri}=\frac{\sigma[2cos\theta\sqrt{D^{2}+\frac{W^{2}}{4}}-W]}{\rho g\frac{WD}{2} Sin\alpha}$ (A.27) |
| --- |

*Curved cross-section:
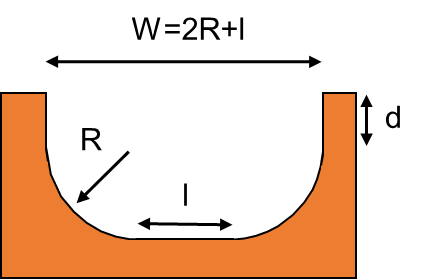
*

σ_sv_ = σ_sl_ + σ_lv_ cos θ (A.28)

F_g_= mg =ρgh[2dR+dl+Rl+0.5πR^2^] (A.35)

F_c_= -dE/dy = σ_lv_[-2R-l+(2d+l+πR)cosθ] (A.34)

dA_sl_ =(2d+l+πR)dy (A.33)

dA_lv_= (2R+l)dy (A.32)

E=σ_lv_(A_lv_-A_sl_cosθ) (A.31)

F_c_= -dE/dy (A.29)

dE=σ_sv_dA_sv_ + σ_sl_dA_sl_+ σ_lv_dA_lv_ (A.30)

Since F_c_=F_g_ $Sin\alpha$, it results:

| $h_{curv}=\frac{\sigma[(\pi R+2d+l)cos\theta-2R-l]}{\rho g\left[ 2dR+dl+Rl+\frac{\pi R^{2}}{2} \right]Sin\alpha}$ (A.36) |
| --- |

*Trapezoidal cross-section:
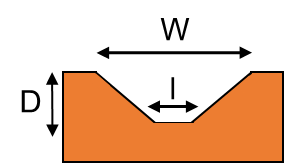
*

σ_sv_ = σ_sl_ + σ_lv_ cos θ (A.37)

F_g_= mg =ρg((l+W)Dh/2) (A.44)

F_c_= -dE/dy = σ_lv_[cosθ(l+2√(𝐷^2+(𝑊-l)^2/4))-W] (A.43)

dA_sl_ =[l+2√(𝐷^2+(𝑊-l)^2/4)]dy (A.42)

dA_lv_= Wdy (A.41)

E=σ_lv_(A_lv_-A_sl_cosθ) (A.40)

F_c_= -dE/dy (A.38)

dE=σ_sv_dA_sv_ + σ_sl_dA_sl_+ σ_lv_dA_lv_ (A.39)

Since F_c_=F_g_ $Sin\alpha$, it results:

| $h_{trap}=\frac{\sigma[(l+2\sqrt{D^{2}+\frac{{(W-l)}^{2}}{4}})cos\theta-W]}{\rho g\frac{(l+W)D}{2} Sin\alpha}$ (A.45) |
| --- |

*Elliptical cross-section:
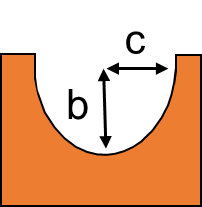
*

σ_sv_ = σ_sl_ + σ_lv_ cos θ (A.46)

F_g_= mg ρ_l_g(πb^2^εh/2) (A.53)

dA_sl_ =2bE(√1- ε^2^)dy (A.51)

dA_lv_= 2bεdy (A.50)

E=σ_lv_(A_lv_-A_sl_cosθ) (A.49)

F_c_= -dE/dy (A.47)

dE=σ_sv_dA_sv_ + σ_sl_dA_sl_+ σ_lv_dA_lv_ (A.48)

F_c_= -dE/dy = σ_lv_[(2bE(√1- ε^2^))cosθ-2bε] (A.52)

Since F_c_=F_g_ $Sin\alpha$, it results:

| $h_{ellip}=\frac{\sigma[(2bE(\sqrt{1-\varepsilon^{2}}))cos\theta-2b\varepsilon]}{\rho g\left[ \frac{\pi\varepsilon b^{2}}{2} \right]Sin\alpha}$ (A.54) |
| --- |

An aspect ratio is defined for the elliptical micro-groove as:

$0\leq\varepsilon\equiv\frac{c}{b}\leq1$ (A.55)

For an elliptical micro-groove, the cross-sectional area and the perimeter are:

$\left\{\begin{matrix} A=\pi bc \\ P=4bE\left(\sqrt{1-\varepsilon^{2}}\right) \end{matrix}\right.$ (A.56)

Where, $E\left(x\right)=\int_{0}^{\pi/2} \sqrt{1-x^{2}\sin^{2}t} dt$is the complete elliptic integral of the second kind.

**Appendix II**

In this Appendix, a step-by-step approach is given to get the non-dimensional capillary rise equation for different cross-sections.

*Rectangular cross-section:*

| $h_{rec}=\frac{\sigma[\left( 2D+W \right)cos\theta-W]}{\rho gDW Sin\alpha}$ | (A.57) | |
| --- | --- | --- |
| P_w_=2D+W and A_c_=DW | |

$\frac{\rho gh Sin\alpha}{\sigma}=$ $\frac{P_{w} cos\theta-W}{A_{c}}=\frac{P_{w} cos\theta}{A_{c}}-\frac{W}{A_{c}}$ (A.58)

$h^{*}=\frac{\rho gh Sin\alpha}{\sigma}*\frac{A_{c}}{P_{w}}=$ $cos\theta-\frac{W}{P_{w}}$ (A.59)

Where, L $=\frac{W}{P_{w}}$ is a characteristic length scale.

*Cylindrical cross-section:*

$h_{cyl}=\frac{\sigma[(\pi R+2d)cos\theta-2R]}{\rho g\left[ 2dR+\frac{\pi R^{2}}{2} \right]Sin\alpha}$ (A.60)

P_w_=$\pi R+2d$ and A_c_=$2dR+\frac{\pi R^{2}}{2}$

$\frac{\rho gh Sin\alpha}{\sigma}=$ $\frac{P_{w} cos\theta-W}{A_{c}}=\frac{P_{w} cos\theta}{A_{c}}-\frac{W}{A_{c}}$ (A.61)

$h^{*}=\frac{\rho gh Sin\alpha}{\sigma}*\frac{A_{c}}{P_{w}}=$ $cos\theta-\frac{W}{P_{w}}$ (A.62)

Where, L $=\frac{W}{P_{w}}$ is a characteristic length scale.

*Triangular cross-section:*

$h_{tri}=\frac{\sigma[2cos\theta\sqrt{D^{2}+\frac{W^{2}}{4}}-W]}{\rho g\frac{WD}{2} Sin\alpha}$ (A.63)

P_w_=$2\sqrt{D^{2}+\frac{W^{2}}{4}}$and A_c_=$\frac{WD}{2}$

$\frac{\rho gh Sin\alpha}{\sigma}=$ $\frac{P_{w} cos\theta-W}{A_{c}}=\frac{P_{w} cos\theta}{A_{c}}-\frac{W}{A_{c}}$ (A.64)

$h^{*}=\frac{\rho gh Sin\alpha}{\sigma}*\frac{A_{c}}{P_{w}}=$ $cos\theta-\frac{W}{P_{w}}$ (A.65)

Where, L $=\frac{W}{P_{w}}$ is a characteristic length scale.

*Curved cross-section:*

$h_{curv}=\frac{\sigma[(\pi R+2d+l)cos\theta-2R-l]}{\rho g\left[ 2dR+dl+Rl+\frac{\pi R^{2}}{2} \right]Sin\alpha}$ (A.66)

P_w_=$\pi R+2d+l$and A_c_=$2dR+l\left( d+R \right)+\frac{\pi R^{2}}{2}$

$\frac{\rho gh Sin\alpha}{\sigma}=$ $\frac{P_{w} cos\theta-W}{A_{c}}=\frac{P_{w} cos\theta}{A_{c}}-\frac{W}{A_{c}}$ (A.67)

$h^{*}=\frac{\rho gh Sin\alpha}{\sigma}*\frac{A_{c}}{P_{w}}=$ $cos\theta-\frac{W}{P_{w}}$ (A.68)

Where L $=\frac{W}{P_{w}}$ is a characteristic length scale.

*Trapezoidal cross-section:*

$h_{trap}=\frac{\sigma[(l+2\sqrt{D^{2}+\frac{{(W-l)}^{2}}{4}})cos\theta-W]}{\rho g\frac{(l+W)D}{2} Sin\alpha}$ (A.69)

P_w_=$l+2\sqrt{D^{2}+\frac{{(W-l)}^{2}}{4}}$ and A_c_=$\frac{(l+W)D}{2}$

$\frac{\rho gh Sin\alpha}{\sigma}=$ $\frac{P_{w} cos\theta-W}{A_{c}}=\frac{P_{w} cos\theta}{A_{c}}-\frac{W}{A_{c}}$ (A.70)

$h^{*}=\frac{\rho gh Sin\alpha}{\sigma}*\frac{A_{c}}{P_{w}}=$ $cos\theta-\frac{W}{P_{w}}$ (A.71)

Where, L $=\frac{W}{P_{w}}$ is a characteristic length scale.

*Elliptical cross-section:*

$h_{cyl}=\frac{\sigma[(\pi R+2d)cos\theta-2R]}{\rho g\left[ 2dR+\frac{\pi R^{2}}{2} \right]Sin\alpha}$ (A.72)

P_w_=$2bE(\sqrt{1-\varepsilon^{2}})$ and A_c_=$\frac{\pi\varepsilon b^{2}}{2}$

$\frac{\rho gh Sin\alpha}{\sigma}=$ $\frac{P_{w} cos\theta-W}{A_{c}}=\frac{P_{w} cos\theta}{A_{c}}-\frac{W}{A_{c}}$ (A.73)

$h^{*}=\frac{\rho gh Sin\alpha}{\sigma}*\frac{A_{c}}{P_{w}}=$ $cos\theta-\frac{W}{P_{w}}$ (A.74)

Where, L $=\frac{W}{P_{w}}$ is a characteristic length scale.
